# The Role of Dental Occlusion and Neuromuscular Behavior in Professional Ballet Dancers’ Performance: A Pilot Study

**DOI:** 10.3390/healthcare9030251

**Published:** 2021-03-01

**Authors:** Henri Didier, Fausto Assandri, Francesca Gaffuri, Davide Cavagnetto, Andrea Abate, Michele Villanova, Carlo Maiorana

**Affiliations:** 1 Department of Biomedical, Surgical and Dental Sciences, School of Dentistry, University of Milan, 20100 Milan, Italy; hdidier@email.it (H.D.); francesca.gaffuri@unimi.it (F.G.); davide.cavagnetto@gmail.com (D.C.); andreabate93@gmail.com (A.A.); carlo.maiorana@unimi.it (C.M.); 2 Fondazione IRCCS Cà Granda, Ospedale Maggiore Policlinico, 20100 Milan, Italy; 3 Areadance Milano, Affiliata ASI Riconosciuta dal CONI, Viale Cassinis 33, 20100 Milan, Italy; m65villanova@gmail.com

**Keywords:** well-being of the oral cavity, oral rehabilitation, occlusion, dance, temporomandibular joint, oral pathologies treatment

## Abstract

Clinical practice and some scientific evidence seem to suggest that there is some kind of relationship between the components that form the postural chain. For professional dancers, good posture and balance are essential. The aim of the present retrospective study is to evaluate whether gnathological treatment could have an impact on the postural balance and sports performance of professional ballet dancers. Electromyographic (EMG) data and balance tests were recorded before and after six months of treatment with a customized occlusal splint. Twenty athletes were examined during ballet exercises in terms of balance and speed of execution by two experienced clinicians. The results showed statistically significant changes for all EMG tests carried out and the Flamingo Balance Test. It appears that the use of a customized occlusal device improved neuro-muscular coordination and the overall performance of dancers.

## 1. Introduction

Many researchers have suggested a relationship between the stomatognathic system and body muscles located far away from the mouth [1,2,3].

In 1895, the investigation proposed by Daniel David Palmer was the first one based on mouth kinesiology and was followed by many other studies that developed kinesiology’s approach to the temporomandibular joint (TMJ).

Gelb, in 1977, observed a better efficiency of competitive athletes wearing an occlusal removable mandibular Orthopedic Repositioning Appliance (MORA) [4,5,6].

Research on the power of occlusion in athletes in relation to non-athletes found that the maximum force in occlusion was considerably higher in athletes, highlighting the relationship between the masticatory muscles implicated in occlusion and the force produced by the spine’s postural muscles [7].

A recent literature evaluation taking into consideration the connection between occlusion, TMJ, and sport performance presented a few articles on this topic, but that had a limited reliability, as they were not randomized clinical trials or meta-analyses [8,9]. Some of the latest research has found muscular and postural modifications by means of kinesiology analysis, but it did not take into consideration reliable tests such as the Messerman or stomatognathic reset tests [10].At the same time, the connection between the stomatognathic system, posture, and musculature has achieved value in sports [3,11,12,13,14,15].Competitive athletes exercise at the highest levels of their physical limits, stressing their body more extensively and rapidly, permitting better and complete investigations of the probable reciprocal influences of these correlations [16].

Dance performance is a complex combination of art and sport that affects the musculoskeletal system in terms of strength, balance, and flexibility. Ballet requires specific movements, static and dynamic balance, and the precise coordination of multi-joint limbs with postural control to achieve an optimum aesthetic level of dance performance and to reduce the risk of musculoskeletal injuries [17].

In classical ballet, the execution of extreme movements on small bases of support requires great balance and specific equilibrium exercises [18,19,20,21,22,23]. Ballet training has a positive effect on the postural stability in standing, as reported by Giboin et al. [24]. Michalska et al. [25] reported that professional dancers had better postural control in comparison to non-dancers while performing simple motor tasks. Visual information was also shown to play an important role in the process of maintaining a stable position of the dancer’s body [26]. Simmons et al. [27] stated that dancers demonstrated better balance due to more consistent neuromuscular responses, a higher proprioceptive sensitivity, and greater muscle activation. Golomer et al. [26] suggested that one of the reasons why professional dancers were less dependent on vision for dynamic postural control was that dance training presumably shifts the sensorimotor dominance from vision to proprioception. Currently, scientific literature requires more concrete evidence on the association between occlusion, the mandible, posture, and the musculoskeletal system. For this reason, it would be desirable to apply scientific methods to study athletes [28,29]. At the same time, however, the recruitment of these subjects, and their continued commitment, does not allow constant monitoring or easy availability [30,31,32].

Since it is known that there is a correlation between the different parts of the body, an assessment of the neuro-muscular balance of the cranio-mandibular district and its possible involvement in postural balance and ballet exercises has been conducted. The purpose of this study was to assess whether a change in occlusal contacts by means of a temporary occlusal device called an orthotic is able to improve the ballet dancers’ performance in terms of the expression of power, management of the construction and evolution of the suspension in the step, the topographical aspect of the movement, and the precision of performing certain dance steps.

## 2. Materials and Methods

The sample was selected from a professional ballet school in Milan, Italy. All participants, or their parents, were informed about the purpose of the study and signed a written consent form. All the procedures involving human participants were carried out in accordance with the ethical standards of the institutional and/or national research committee and with the World Medical Association Declaration of Helsinki of 1975, revised in Tokyo in 2004. The study protocol was approved by the Ethical Committee of the Fondazione IRCCS Ca’ Granda, Ospedale Maggiore, Milan (Prot. 13/07/2017).

Inclusion criteria were a Caucasian ethnicity; good general health; patients with TMJ involvement at the baseline assessed as negative according to RDC-TMD [33]; no previous orthodontic treatment; an absence of parafunctional habits (grinding and clenching); and Decayed, Missing, Filled Teeth (DMFT) equal to 0. A group of twenty (16 females and 4 males) professional ballet dancers with a mean age of 18.4 ± 2 years (range of 14–21 years old) were included in the study. None of them were injured at that time and all were performing on a regular basis. They practiced five to six days per week, three to eight hours per day. The following measurements and data were collected for all ballet dancers, at the Department of Biomedical, Surgical and Dental Sciences, University of Milan, Fondazione IRCCS Ca’ Granda, Ospedale Maggiore Policlinico, between September 2017 and May 2018: The Flamingo Balance Test and kinesiographic and electromyographic (EMG) tests were recorded.

### 2.1. Electromyography and Kinesiographic Assessment

The electromyographic (EMG) and kinesiographic (KG) tests were performed for masseter, anterior temporalis, sternocleidomastoid muscle, and upper trapezius muscles at the habitual rest position and during a two-seconds maximal clench on natural dentition and cotton rolls, in order to determine and compare the muscular efficiency and the correct relationship between masseter and anterior temporal muscles at different occlusal positions (scan 9 and scan 11) [34]. The K7 Diagnostic System (EMG electromyograph and CMS kinesiograph) and the J4 trans-electrical nerve stimulation (TENS) unit (Myotronics, Normed Inc. Tukwila, Washington) with Myotrode SG mono-polar electrodes were used for this electrophysiological study.

The electrodes for the EMG recording were applied in the midline of the belly of the muscle between the nearest innervation zones and myotendinous junction. In this location, the EMG signal with the greatest amplitude is detected.

The superficial beam of the masseter was investigated with the arrangement placed at the mandibular angle in a straight line from the edge, with the last electrode corresponding to the mandibular angle. The positions of the electrodes in the muscle will be specified below as the distance from the mandibular angle, normalized with respect to the mandibular angle distance from the edge. Two reference lines were considered for the non-invasive evaluation of the anterior temporal muscle. The first was the straight line that passed between the mandibular angle and the condylar head, rotated forward with an inclination of 20°. The second was the line tangent to the ear of the auricle and passing through the canthus. The array was placed along the first line, with the last electrode at the intersection of the two reference lines. The position of the electrode in the previous temporal will be indicated below as the distance from the intersection of the two selected lines [35,36,37].

The recording of the muscular activity began by inviting the subject to remain with their jaw in a resting position and to subsequently clench on cotton rolls for 2 s and then return to the rest position (scan 11). The kinesio-electromyographic analysis of the maxilla–mandibular relationship was performed before and after 45 min of TENS, in order to identify the physiological rest position of the mandible corresponding to the minimal tonus of both the elevator and depressor muscles. The TENS used for this investigation was characterized by a pulse duration of 500 μsec, a frequency of 40 pulses per minute, and a variable intensity from 0 to 25 mA, depending on the threshold of each individual patient. The electrodes for the neurostimulation were applied on the coronoid notch of the mandible at the V° and VII° pairs of cranial nerves’ level [38].

Kinesiographic examinations were used to define mandibular retrusion after TENS (scan 5) or deviation on the sagittal and frontal plane (scan 4/5) and atypical swallowing (scan 20) [39,40]. An occlusal device, called an orthotic, was customized directly in the athletes’ mouth, after 45 min of TENS, which allowed us to reposition it and obtain a physiological mandibular position following the neuromuscular trajectory obtained by TENS on the sagittal and frontal plan. The importance of neuromuscular factors in the onset of temporomandibular disorder (TMD) has led to the development of specific clinical and instrumental evaluations, which should be taken into consideration to produce a removable mandibular orthotic device. An orthosis is a mobile appliance that needs to be continuously worn by the subjects in order to improve the mandible stabilization, as well as physiological functioning of the masticatory muscles. The oral device used in the present study was made of acrylic resin and was a removable mandibular orthotic device, with easy insertion and extraction for minimizing stress and pressure on the teeth.

An occlusal splint was considered as the occlusal device most suitable for the study since it is applied to the mandible during ballet and dancer’s exercises to evaluate the influence of occlusion on the physical activity and its effect on the skeletal muscle performance. All of the athletes were encouraged to wear it at least 20/22 h/day to evaluate whether there was an improvement in performance both subjectively and objectively. All dancers were re-evaluated with the same equipment and methods six months after the use of this device and they were filmed and timed throughout the duration of the exercises to evaluate their improvements in balance and control.

After the orthotic therapy, questionnaires were also completed by the athletes, in order to understand their experience and to verify for how long they really used the occlusal device during training and exercises of classical ballet.

### 2.2. Flamingo Balance Test

Postural stability was tested by the Flamingo Balance Test, which is a total body exercise that assesses the strength of the leg, pelvic, and trunk muscle, as well as dynamic balance. The order of the tests was randomized for each participant.

The procedure consisted of standing in a unipedal stance with one single preferred leg. The free leg was flexed back, with the back of the foot being grasped by the corresponding hand, and the participant remained balanced on one leg like a flamingo dancer. The position was tested barefoot and with ballet shoes while keeping the eyes opened and barefoot while keeping the eyes shut, counting the number of falls in 60 s of balancing in each of the three conditions. The test was interrupted whenever the subject lost balance (or when the hand released the foot or when the ground was touched with any part of the body).

### 2.3. Statistical Analysis

The following variables were collected for each subject before and after six months of treatment with an intraoral splint: Free-way space (FWS); atypical swallowing (A.S.); masseter muscle (MM); anterior temporal muscle (AT); sternocleidomastoid muscle (SCOM); trapezius (TR); muscular relationship recruitment, which is the ratio between the omolateral masseter muscle and the temporalis anterior (MM/TA); and the Flamingo Balance Test while barefoot, both with eyes opened and eyes shut and while using ballet shoes with the eyes opened. The data distribution for all variables was assessed with the Shapiro–Wilk test. Pairwise comparisons were performed with a paired t-test for continuous normally distributed variables, with a Wilcoxon signed rank test for continuous not-normally distributed variables and with a binomial sign test for binomial data.

## 3. Results

Eighteen dancers showed an increase in muscular activity for masseter and anterior temporal muscles. Twelve dancers showed increased values for sternocleidomastoid muscle and trapezius muscles.

Five ballet dancers presented asymmetrical EMG values (scan 11) at the baseline for masseter muscles and seven subjects exhibited asymmetrical activation of the anterior temporal muscles. Atypical swallowing was found in six athletes, and a mandibular retrusion of 0.3–3.7 mm was corrected with the application of the gnathological splint for all the dancers that participated in our study.

The results of the Flamingo Balance Test, collected for the assessment of balance, showed an improvement in the ability to maintain balance for 60 s after the application of the intraoral splint.

After the six months of splint management, the new EMG examination revealed a statistically significant improvement of their myoelectric signal for both scan 9 and 11. Only three dancers revealed no changes in asymmetry of muscular activation. Comparing the electromyographic values of the trapezius muscles with the postural assessment, no significant correlation appeared between EMG values and shoulder alignment.

EMG and kinesiographic values, along with Flamingo Balance Test results before and after intraoral splint therapy, are presented in Table 1.

## 4. Discussion

The aim of the current investigation was to evaluate whether the application of an occlusal splint could result in any modifications in the professional classic dancers’ postural balance. Proprioception plays an essential role in postural adjustments and provides the muscular system with information on the position of the different parts of the body. These receptors’ position in the stomatognathic system are located in the periodontal ligament, muscles, tongue, and temporomandibular joint. The central nervous system receives all this information, integrating it and thus creating different postural and behavioral responses. If an occlusal imbalance occurs, this dysfunction may lead to muscle decompensation in an attempt to compensate for the mandibular malposition with repercussions for posture. The balance of the orofacial musculature, obtained and maintained using an intraoral splint, allows balance to be transferred to the whole body. The practice of dance is based on balance and good posture: It is essential for ballet dancers to implement particular exercises to be able to perform complex movements on very small support bases, as indicated by Rangel et al. [18].

Professional dancers have more advanced postural control than non-dancers in performing common motor exercises, as demonstrated by Giboin et al. [24] and Michalska et al. [25]. We therefore decided to evaluate whether an improvement in occlusal contacts, implemented through a temporary customized occlusal device, could have a positive effect on the performance of professional dancers.

The use of the electromyographic examination was of fundamental importance for the realization of this study [41]. The EMG evaluation of muscular activities allowed us to evaluate any functional anomalies and possible muscular pathologies, as reported by Didier et al. [42]. In this study, the EMG examination was performed for the masseter, anterior temporal, sternocleidomastoid, and superior trapezius muscles, both in the resting position and during the clamping of the dental elements. Muscle activity analysis was evaluated by applying TENS in correspondence with the coronoid notch of the participants’ mandible, at the level of V and VII cranial nerves [43,44,45,46].

The electromyographical examination underlined asymmetries of muscular activities and mandibular retrusion for the majority of the dancers. For this reason, the sample was rehabilitated with the application of a mandibular repositioning device called an orthotic. Data about postural assessment are probably attributable to the fact that any misalignments and inclinations of the lines observed were due to ascending and not occlusal-derived postural problems. The reduction of head inclination found for three dancers can be attributed to the corrective action of the orthotic in subjects with occlusal instability.

The Flamingo Balance Test showed statistically significant improvement after six months of splint treatment. The EMG examination also showed a statistically significant improvement, both for the muscles of the stomatognathic region and for the muscles intervening in the posture of the skull.

The use of the intraoral splint allows the muscles of the neck and upper shoulders to immediately relax, making mobility much more effective. The stomatognathic system connects the skull to the shoulder girdle and is the starting point of numerous postural anterior and posterior chains that connect the mandible to the hyoid bone, sternum, and clavicle, and the neurocranium to the splanchocranium, as stated by Cuccia et al. [10,47,48,49]. If tension occurs at these levels, the human body may activate compensatory mechanisms. An imbalanced occlusion can lead to a displacement of the mandible, and this incorrect position can promote muscular decompensation of the whole body to various extents, in order to try to compensate for the mandibular malposition. These events clearly have repercussions for posture.

Travell et al. [50,51,52,53] identified functional mechanisms that are at the basis of postural alterations linked to asymmetries of the lower limbs, minor hemi-pelvis, and postural attitudes or traumas that, by causing a shortening of the muscle fibers, are able to activate the appearance of trigger points.

The results obtained from the study therefore demonstrate that the use of an individual occlusal device, employed to improve dental occlusion and thus restore and improve the muscular balance of the orofacial region, leads to a statistically significant improvement in muscle balance of the whole body and thus postural balance. It could be assumed that after six months of orthotic treatment, the postural balance improved with the postural occlusion. The more correct the posture, the more the work of the muscles will be favored; postural defects do not allow power to be expressed correctly.

The ballet dancer, in sports, represents the only example for which balance is essential for aesthetics. Analyzing the various and possible skeletal types, as already demonstrated in the literature (CIT), a skeletal class II individual according to Steiner et al. is considered to be ‘more disadvantaged’ in terms of posture and balance than a class III subject. Evaluating the sample of dancers in this study, it was shown that the class II subjects tended to bring the head back in hyperextension and the shoulders forward, increasing the cervical lordosis and decreasing the anterior podalic support, with flattening of the cervical lordosis, straightening of the column, and anteriorization of the pelvis. Skeletal class III individuals tended to bring their heads forward, favoring an anterior podalic support that causes less postural problems. Previous literature has identified functional mechanisms that underlie postural alterations, that is, related to asymmetries of the lower limbs, smaller emi-bacino, postural attitudes, or trauma that, by causing a shortening of the muscle fibers, are able to activate the appearance of trigger points [50,51,54]. In reality, the authors mentioned have never had precise investigation means capable of quantifying postural alterations of the jaw at their disposal. The chewing and neck muscles are connected to muscles of the whole body: Obviously, asymmetry or a malfunction of these muscles causes, in most cases, a chain effect, with the alteration of the muscular work of the other muscles of the body. The initial malfunction is thus amplified.

## 5. Conclusions

The influence of occlusion on posture and the correlation between occlusal variation and balance changes have been demonstrated in this study. Postural balance and classical ballet performance could be improved with the application of the orthotic, in subjects who showed asymmetrical muscle activation.

The gnathological splint may have no effect in cases with autonomous and adaptive body compensation for the postural imbalance. Further studies evaluating the effects of the orthotic appliances in professional ballet dancers with longer assessment periods are needed.

## Figures and Tables

**Table 1:** Statistical comparison of the electromyographic (EMG) and kinesiographic values before and after trans-electrical nerve stimulation (TENS) and orthotic treatment.

Variables	T1			T2			*p* Value
	Mean ± SD	Median	IQR	Mean ± SD	Median	IQR	
FWS (Scan 3)	1.06 ± 0.77 ^§^	0.75	0.6	3.33 ± 1.71	3.35	2.7	<0.001 **
R-MM (Scan 9)	2.53 ± 0.59 ^§^	2.25	1.1	2.14 ± 0.20 ^§^	2.05	0.3	0.024 *
L-MM (Scan 9)	2.51 ± 0.76	2.40	0.9	2.08 ± 0.35 ^§^	2.10	0.4	0.040 *
R-TA (Scan 9)	2.85 ± 0.60	2.80	1.0	2.33 ± 0.58 ^§^	2.50	0.5	0.038 *
L-TA (Scan 9)	2.69 ± 0.60	2.75	1.1	2.07 ± 0.50	2.00	0.7	0.005 *
R-SCOM (Scan 9)	2.39 ± 0.56 ^§^	2.10	0.9	2.59 ± 0.34 ^§^	2.50	0.4	0.063
L-SCOM (Scan 9)	2.36 ± 0.62	2.20	1.2	2.71 ± 0.37 ^§^	2.80	0.2	0.007 *
R-TR (Scan 9)	2.41 ± 0.70 ^§^	2.10	1.2	2.68 ± 0.42 ^§^	2.50	0.5	0.021 *
L-TR (Scan 9)	2.54 ± 0.77 ^§^	2.10	1.4	2.84 ± 0.47 ^§^	2.80	0.2	0.032 *
A.S. ^#^ (Scan 20)	30%			0%			0.031 *
MM/TA ^#^ (Scan 11)	30%			0%			<0.001 **
FBT barefoot	57.75 ± 5.50	60	0	60 ± 0.0	60	0	0.083
FBT ballet shoes	60 ± 0.0	60	0	60 ± 0.0	60	0	1
FBT eyes closed	21.30 ± 22.03	11.50	33	28.90 ± 20.07	20	38	0.013 *

Legend: FWS, free-way space; A.S., atypical swallowing; R/L MM, right/left masseter muscle; R/L AT, right/left anterior temporal muscle; R/L SCOM, right/left sternocleidomastoid muscle; R/L Tr, right/left trapezius; MM/AT, muscular relationship recruitment (masseter muscle/temporalis anterior); FBT, Flamingo Balance Test. * *p* < 0.05; ** *p* < 0.01; ^§^ not normally distributed data. Wherever not specified, data are normally distributed; ^#^ binomial data, where the descriptive statistics are expressed as the percentage of patients presenting the variable and inferential statistics as the signed rank test.

## Data Availability

Data of the present study are available from the corresponding author on reasonable request.
